# Responses to soil pH gradients of inorganic phosphate solubilizing bacteria community

**DOI:** 10.1038/s41598-018-37003-w

**Published:** 2019-01-10

**Authors:** Bang-Xiao Zheng, Ding-Peng Zhang, Yu Wang, Xiu-Li Hao, Mohammed A. M. Wadaan, Wael N. Hozzein, Josep Peñuelas, Yong-Guan Zhu, Xiao-Ru Yang

**Affiliations:** 1 Key Laboratory of Urban Environment and Health, Institute of Urban Environment, Chinese Academy of Sciences, Xiamen, 361021 PR China; 2 University of Chinese Academy of Sciences, Beijing, 100049 PR China; 3 Consejo Superior de Investigaciones Científicas (CSIC), Global Ecology Unit, Centre for Ecological Research and Forestry Applications (CREAF)–CSIC–Universitat Autonoma de Barcelona (UAB), Bellaterra, 08193 Barcelona, Catalonia Spain; 4 CREAF, Cerdanyola del Vallès, 08193 Barcelona, Catalonia Spain; 5 State Key Laboratory of Biocontrol, Key Laboratory of Biodiversity Dynamics and Conservation of Guangdong Higher Education Institutes, College of Ecology and Evolution, Sun Yat-sen University, Guangzhou, 510275 PR China; 6 Key Laboratory of Soil Environment and Pollution Remediation, State Key Laboratory of Soil and Sustainable Agriculture, Institute of Soil Science, Chinese Academy of Sciences, Nanjing, 210008 PR China; 7 Department of Plant and Environmental Sciences, University of Copenhagen, Frederiksberg, 1871 Denmark; 8 Bioproducts Research Chair, Zoology Department, College of Science, King Saud University, Riyadh, 11451 Saudi Arabia

## Abstract

Soil pH is commonly considered a dominant factor affecting the function of microbiota. Few studies, however, have focused on communities of bacteria able to solubilize inorganic phosphate (iPSB), which are important for the mobilization of soil phosphorus (P), because finding an effective method to assess the abundance and diversity of iPSB communities is difficult. We used a newly reported method of database alignment and quantified the gene *pqqC* to analyze the compositions of iPSB communities from five soils with pH gradients ranging from 4 to 8. The iPSB community structure differed significantly between these soil types. Among iPSB community, *Bacillus* was the dominant genus, followed by *Arthrobacter* and *Streptomyces*. A redundancy analysis indicated that soil pH was the most important of 15 soil factors and their pairwise interactions, accounting for 5.12% of the variance. The abundance of the iPSB communities increased with pH within the gradients which was confirmed by experimental adjustment of pH, suggesting that the defect P status in high pH soil was speculated as the driving force of iPSB community population. Our study demonstrated the dominant role of soil pH on the iPSB community, which may contribute to the understanding the possible mechanism of microbial P mobilization for better improvement of P use-efficiency.

## Introduction

Phosphorus (P) is a non-renewable resource in nature^1,2^, but is now majorly used for P fertilizer production^3^. The applied P is readily immobilized soil fixation and the available P is consequently decreased for crop growth^4^. This available P defect induce more input of P fertilization, which causes P residue accumulations. The negative effect of P residues may persist for many years, including decreasing the amount of labile P and thus P availability^5^. Finding a way to sustainably preserve P resources is thus of great importance.

Microorganisms could positively assist plant P uptake, especially bacteria that can solubilize inorganic phosphate–inorganic phosphate solubilizing bacterias (iPSB). The common and powerful iPSB included *Pseudomonas*, *Bacillus*, *Burkholderia*, *Rhizobium* and *Actinomycetes* spp^6^. iPSB communities can effectively solubilize fixed P into bioavailable forms and prevent the released P from being immobilized again^6,7^. iPSB under conditions of P deficiency can donate protons to the external environment, acidifying it for P release^6,8^. iPSB can also secrete organic anions, which weaken strong bonds with metal ions by competitive binding^7,9^. iPSB communities harbor various iPSB species with different P-solubilizing abilities, including different types and amounts of extruded organic anions, which may be affected by environmental changes. To study the key environmental factors affecting iPSB community structure is important.

Generally, the distribution of total bacterial community would be affected by some common environmental factors, such as temperature and oxygen availability. Soil pH is widely considered as a universal indicator of the structural features of bacterial communities and is closely associated with populations of soil microbial communities^10–14^. Microbial communities with specific functions may also be affected by pH gradients. The compositions of ammonia-oxidizing archaeal and bacterial communities, for example, have distinct lineages within the pH gradient in acidic and neutral soils^15^. The unique pH gradient at the Hoosfield acid strip (United Kingdom), which is originally acidic and well drained to moderate, is responsible for shifts in the bacterial and fungal communities and for functional redundancy in carbon (C) mineralization^16^. An analysis of genes associated with the regulation of nitrogen (N) fluxes found that artificially altering soil pH strongly affected the potential denitrifying activity and composition of a denitrifying microbial community^17^. However, whether and how the soil pH, an important driving force of P mobilization, would have an impact on iPSB communities were needed an investigation.

Apart from the common environmental factors, the bacterial community with a specific function may be shaped by some special physical or chemical characteristic. For example, the distribution of ammonia-oxidizing archaea (AOA) community under the water is dependent by sulfide and phosphate concentration^18^. The sulfide was reported to be involved in the expression regulation of one unique AOA gene for 3-hydroxypropionate cycle and the phosphate level was believed to be a limiting factor for the survival of AOA community. The community abundance and structure of soil and sediment methane oxidizer was specifically restricted by N concentration^19^. Furthermore, the community distribution of N-cycling members, including nitrate reducers and nitrifiers, could be patterned by manganese availability and land use, respectively^20^. The regulation of iPSB survival could be attributed to some universal factors for the whole bacteria such as temperature and moisture content;^21^ however, as to the specific affecting factors for iPSB community structure, there is actually scarce investigation.

Estimating iPSB population size and community structure has been hindered by the lack of appropriate techniques. Traditional agar screening underestimates iPSB population sizes, because many microorganisms cannot be cultured. The pyrroloquinoline quinone biosynthesis gene *pqq* is a recognized cofactor for glucose acid and 2-keto-D-glucose acid release^22,23^ and the knockdown of *pqq* gene would reduce the inorganic phosphate solubilizing capacity^24^. The quantification of *pqq* gene may represent, in some extent, the potential size of inorganic P solubilizers. Pyrosequencing of 16 S rRNA gene is massively used in structuring of microbial community. By alignment with iPSB database, the potential iPSB community structure could be obtained. Combining with these two methods, the size and structure of iPSB communities could be estimated^25^. Based on these two methods, a crop field with long-term fertilization was studied and results showed soil pH was the critical factor for structuring iPSB communities^25^. Based on the knowledge above, in this study, we hypothesized that soil pH would strongly affect iPSB community structure. We investigated the structural differences of iPSB communities in five soils with pH ranging from 4 to 8 and analyzed the dominant driving environmental factors. Besides, the pH adjustment was conducted to see the structural change in iPSB community.

## Materials and Methods

### Study sites, soil sampling and pH adjustment

Five sampling sites with pH gradients were chosen (Table S1). Soils P4 and P6 were collected from the agricultural experimental station of South China Agricultural University in Guangdong Province, China (23°16′N, 113°35′E), P5 and P7 were collected from the Yixing (31°26′N, 119°82′E) and Changshu (31°64′N, 120°74′E) experimental stations in Jiangsu Province, respectively^26^, and P8 was collected from farmland in Fengqiu in Henan Province (35°11′N, 114°35′E). Three replicate samples of surface soil (0–15 cm) were collected after wheat harvests in May or June 2014. The soils were then air-dried, passed through a 2.0-mm sieve and stored at 4 °C for further analysis.

Fresh P4 and P8 samples were chosen as representatives of acidic and alkaline soil for pH adjustment, respectively, to examine the changes in iPSB community structure (graphic scheme: Fig. S1). The pHs were adjusted to 4.00, 5.00, 6.00, 7.00 and 8.00 in 20 g of soil by adding autoclaved 1 M NaOH or 0.5 M H_2_SO_4_^27^. These soils were named K4-K8 and A4-A8, respectively, and were then incubated at 28 °C for 7 days. DNA was extracted from all soils for further analysis.

### Analysis of soil physicochemical properties

Soil pH was measured in a dry soil: H_2_O ratio of 1:2.5 (w/v) with a XL60 pH meter (Fisher Scientific, Asheville, USA)^28^. The water content (%) of soil was determined gravimetrically by drying the fresh soils at 105 °C for 16 h. A laser particle-size analyzer (Mastersizer 2000, Malvern Instruments Ltd., Malvern, UK) was used to measure the contents of clay, loam and sand. The soils were sieved through a 0.15-mm mesh, and total C, N and sulfur (S) contents were measured with an elemental analyzer (vario MAX CN, Elementar, Hanau, Germany). NH_4_^+^ and NO_x_^−^ were extracted with 2 M KCl at a soil:KCl ratio of 1:10 and filtered through a 0.22-μm hydrophilic PEPT needle filter (ANPEL, Shanghai, China). The filtrates were analyzed by a flow injection analyzer (QuikChem 8500, Lachat Instruments, USA). Five grams of soil were digested in sulfuric acid^29^ for determining total potassium (K), calcium (Ca), sodium (Na), magnesium (Mg), iron (Fe) and aluminum (Al) contents by inductively coupled plasma optical emission spectrometry (ICP-OES) (Optima 7000DV, Perkin Elmer, Waltham, USA). The contents of total P and available P (AP) were determined using the molybdate-blue method^30^ and sodium bicarbonate extraction^31^, respectively.

### Genomic DNA extraction, high-throughput sequencing and data analysis

Genomic DNA was extracted and sequenced by high-throughput sequencing from all natural and pH-adjusted soils. DNA was extracted from approximately 0.5 g of soil using a FastDNA^®^ Spin Kit for Soil following the manufacturer’s instructions (MP Biomedicals, Santa Ana, USA) and stored at −20 °C. DNA quality was assessed using a NanoDrop ND-2000 spectrophotometer (Thermo Scientific, Waltham, USA), limiting OD_260_/OD_280_ ranges within 1.6–1.8. The DNA concentration was measured with a QuantiFluor^®^ dsDNA system (Promega, Madison, USA) using a multiscan spectrum (SpectraMax M5, Molecular Devices, Shanghai, China).

The 515 F/907 R primer set (515 F: GTGCCAGCMGCCGCGGTAA; 907 R: CCGTCAATTCMTTTRAGTTT) with a 6-mer barcode fused to the reverse primer was used for amplifying bacterial fragments in each sample and subsequent Illumina sequencing^32^. Each 50-μL PCR reaction mixture contained 10 ng of genomic DNA, 1 μL of *Premix Ex Taq* Hot Start Version (TAKARA, Dalian, China), 0.2 μM each primer and 0.1 mg mL^−1^ bovine serum albumin (BSA). The amplification protocol was: initial denaturation at 95 °C for 5 min, 35 cycles of 95 °C for 30 s, 58 °C for 30 s, 72 °C for 30 s and a 5-min extension at 72 °C. The PCR products were purified and quantified using a Universal DNA Purification Kit (TIANGEN, Beijing, China) and the QuantiFluor^®^ dsDNA system, respectively. The purified DNA products were pooled in equal proportions and submitted for sequencing by an Illumina Hiseq.2500 platform (Novogene, Beijing, China).

The QIIME system was used for filtering, processing and analyzing the raw reads^33^, and sequence quality was controlled by default. Operational taxonomic units (OTUs) were clustered with UCLUST clustering at a cutoff of 3% dissimilarity^34^, and representative sequences were retrieved and classified with the RDP classifier^35^.

### iPSB identification and *pqqC* quantification

There are two different methods to quantify the size of iPSB community, database alignment method and qPCR quantification. We jointly used these two methods because both of them were predictive and partly defective that we could not fully trust any one of them until both methods showed the same trend.

iPSB identification based on database alignment has been previously described^25^. The sequencing data were aligned with the iPSB database using Local Blast 2.2.27+ (ftp://ftp.ncbi.nlm.nih.gov/blast/executables/blast+/2.2.27/), and the potential iPSB species were annotated with critical criteria (E < 1 × 10^–10^ and sequence identity > 99%)^25^. A total of 2 082 641 reads of the 16 S rRNA gene were obtained after filtering for quality and removing chimeric reads. These reads were aligned with the iPSB database, and 21 240 sequences with similarities > 99% were accepted as potential iPSB species. The percentages of iPSB in each soil ranged from 0.56 ± 0.07 to 1.90 ± 0.30%, with an average of 1.13 ± 0.56%.

The abundances of *pqqC* and the 16 S rRNA gene were determined by real-time quantitative PCR (qPCR) in triplicate using the Fw/Rv^25^ and F515/R907 (ref) primer sets, respectively. Standard plasmids carrying *pqqC* or the 16 S rRNA gene were constructed by transforming each gene into vectors with a pMD 19-T vector cloning kit (TAKARA, Dalian, China) and were purified with a TIAN prep mini plasmid kit (TIANGEN, Beijing, China). The plasmid concentration was measured by a NanoDrop ND-2000 spectrophotometer (Thermo Scientific, Waltham, USA), and a calibration curve was constructed using 10-fold serial dilutions of standard plasmid DNA. The DNA reaction system for each sample was prepared in triplicate as a mixture of 10 ng of template DNA, 0.3 μM each primer, 1 × SYBR premix Ex *Taq* and 0.1 mg mL^−1^ BSA. The genes were amplified using a LightCycler 480 System (Roche, Basel, Switzerland) and the amplification protocol: initial denaturation at 98 °C for 3 min and 40 cycles of 98 °C for 20 s, 62 °C for 30 s and 72 °C for 30 s. The efficiency of each reaction was 90–110%. The relative abundance of *pqqC* was considered as the ratio of *pqqC* to 16 S rRNA abundance.

### Statistical analyses

Correlation and variance (ANOVA) analyses were conducted using IBM SPSS Statistics 21 (IBM, New York, USA). Principal component analysis (PCA), redundancy analysis (RDA), variance partitioning analysis (VPA) and Monte Carlo permutation test were performed using R studio (version 3.2.3) with the VEGAN package^36^. The figures were generated using SigmaPlot 12.5 (Systat Software, San Jose, USA).

## Results

**Figure 1:**
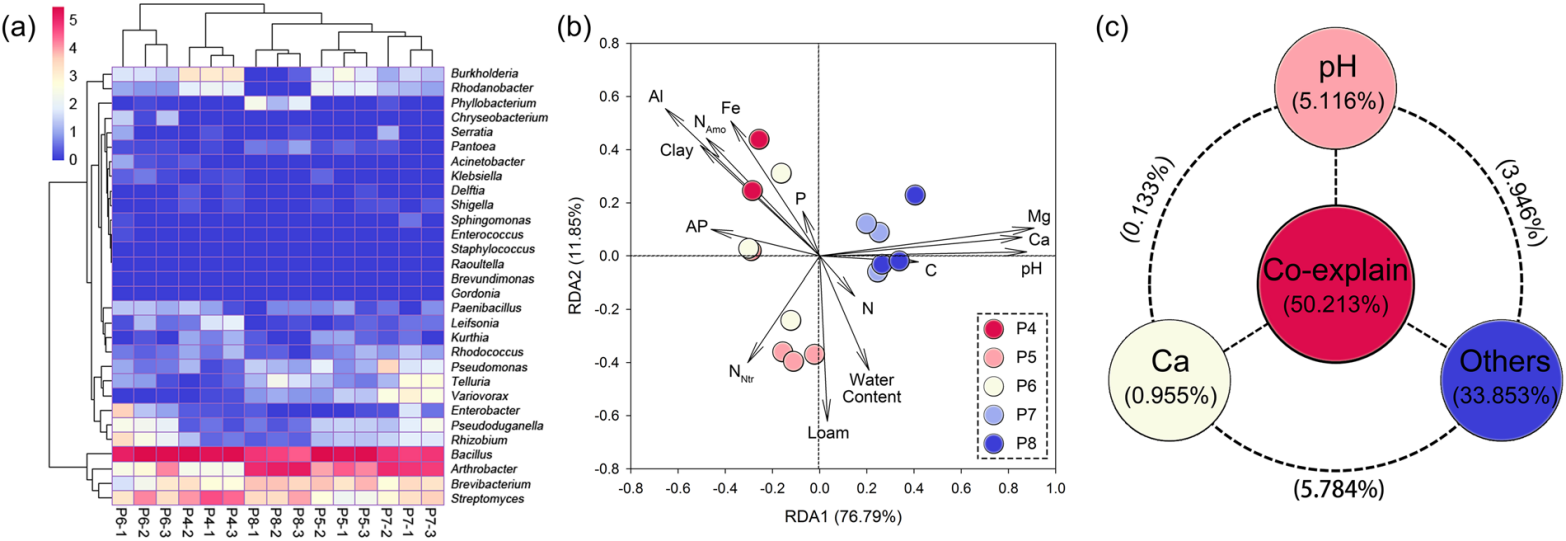
Heatmap, redundancy analysis (RDA) and Variance partitioning analysis (VPA) of iPSB communities. (**a**) The profile of iPSB community distribution based on Bray-Curtis distances and (**b**) the effect of soil chemical properties on iPSB communities in the various soils. The plotted values are natural-logarithm transformations of relative iPSB community abundance. The columns in (**a**) are labeled with soil names and replicate numbers. (**c**) The contribution of soil pH, Ca contents, others (including water, clay, loam and sand contents, total C, N, P, S, K, Na, Mg and Fe contents and AP, ammonium and nitrate or nitrite N contents) and their co-explanation for the structuring of iPSB community.

Five natural soils with pH gradients were showed with different characteristics (a PCA analysis could be found in Fig. S2). By database alignment, the iPSB bacteria was identified and showed differently across pH gradients (Table S2). The pattern of iPSB community structure at the genus level is shown in Fig. 1a. Thirty genera were aligned and *Bacillus* was the dominant genus. The percentages of the total iPSB populations were 57.14 ± 6.58, 59.26 ± 1.35, 58.60 ± 11.48, 34.26 ± 4.40 and 29.75 ± 4.70% in P4, P5, P6, P7 and P8, respectively. Other abundant genera were *Arthrobacter* (20.25 ± 1.19% on average), *Streptomyces* (11.24 ± 0.66% on average) and *Brevibacterium* (6.97 ± 0.32% on average).

The effects of different soil properties on the structuring of iPSB community were analyzed (Fig. 1b,c). The RDA (Fig. 1b) found that K, Na and S contents were redundant and were automatically aliased by the R program, so the structures of the iPSB communities were interpreted using other environmental variables. The total explained proportion RDA1 and RDA2 was 76.79 and 11.85%, respectively (Fig. 1b). pH and Ca and Mg contents were the most important factors affecting the iPSB communities, supported by their significant correlations (*P* < 0.001) in the Monte Carlo permutation tests (Table 1). A clustering analysis based on β diversity clearly grouped the iPSB community structures in each type of soil (Fig. 1a). The contributions of each factor and their pairwise interactions to the compositions of the iPSB communities were quantified, and the percentages of the variance due to single variables or bifactors resolved by a VPA are listed in Table S4. The percent contributions of the most important factors, pH and Ca content, are presented separately in Fig. 1c. The VPA found that 83.92% of the total variance could be attributed to the effects of single or double factors (Table S4). pH accounted for the highest percentage (5.116%) among the 14 environmental variables, and Ca and Mg contents explained 0.995 and 3.754%, respectively, of the variance in iPSB community structure.

**Table 1:** Monte Carlo permutation tests (999 permutations) of the effect of soil chemical properties on iPSB community.

	R^2^	P
pH	0.771	**0.001** ^***^
Water	0.228	0.191
Clay	0.429	**0.028** ^*^
Loam	0.387	0.062
Sand	0.323	0.100
C	0.176	0.310
N	0.045	0.732
N_Amo_	0.428	**0.027** ^*^
N_Ntr_	0.253	0.165
P	0.034	0.818
AP	0.222	0.204
S	0.030	0.846
K	0.198	0.267
Ca	0.742	**0.001** ^***^
Na	0.014	0.929
Mg	0.837	**0.001** ^***^
Fe	0.400	**0.042** ^*^
Al	0.735	**0.003** ^**^

N_Amo_, ammonium N; N_Ntr_, nitrate or nitrite N; ^*^P < 0.05; ^**^P < 0.01; ^***^, *P* < 0.001.

**Figure 2:**
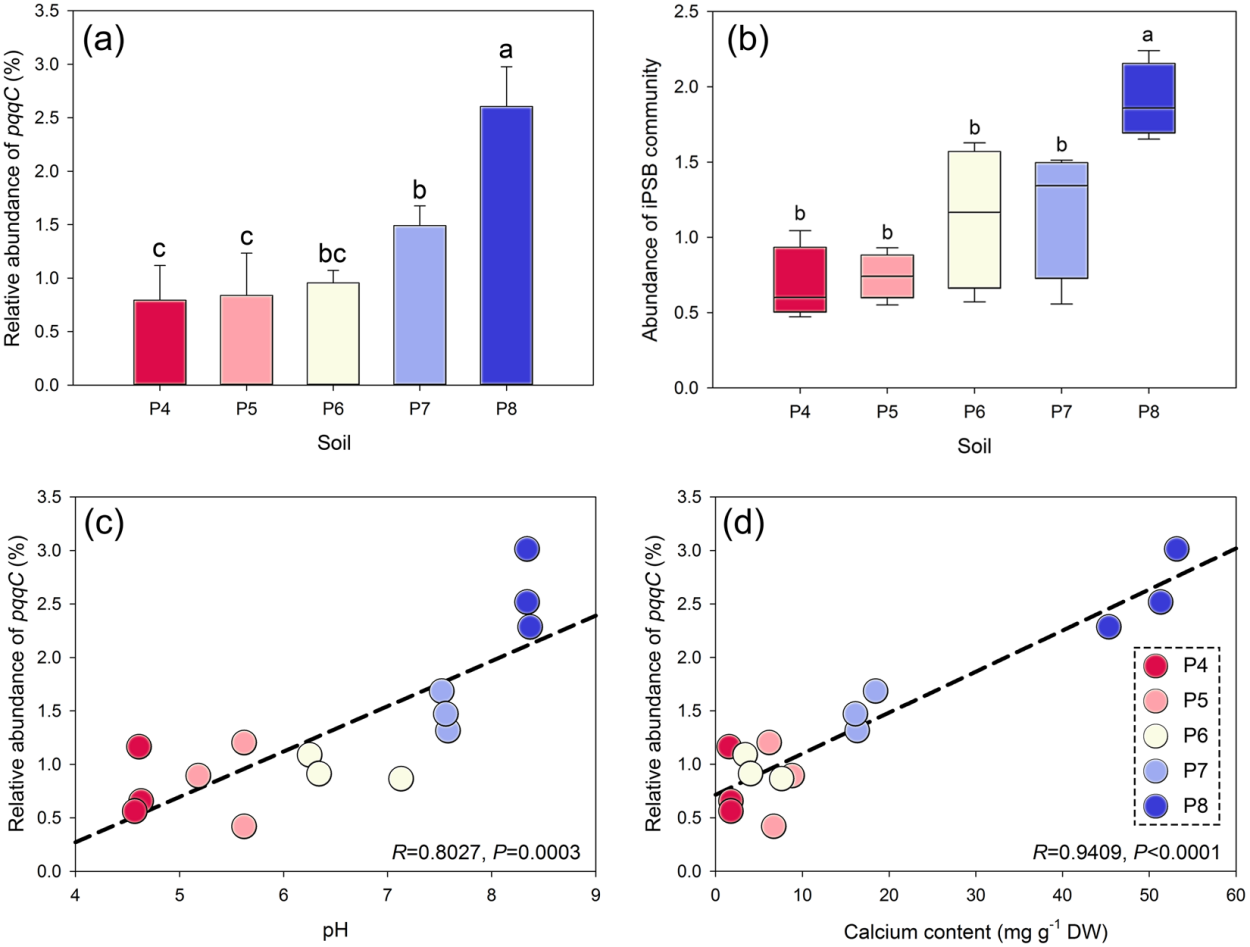
The abundance and the major impact factors of iPSB communities. (**a**) The relative abundance of *pqqC* (%) and (**b**) abundances of the iPSB communities based on database alignment. The effect of soil pH (**c**) and calcium content (**d**) on the relative abundance of *pqqC*. DW, dry weight. Different letters above the bars indicate significant differences at P < 0.05.

Based on the qPCR method, the abundance of iPSB community increased with soil pH gradients (Fig. 2a). The relative abundance of *pqqC* increased with pH, with average relative abundances of 0.79 ± 0.32% (P4), 0.84 ± 0.39% (P5), 0.96 ± 0.12% (P6), 1.49 ± 0.19% (P7) and 2.61 ± 0.37% (P8). This trend was similar to the database alignment results (Fig. 2b). To better illustrate the changes of diversity of iPSB community, Shannon index was utilized to quantify (Fig. S3). The diversity of the iPSB communities was the highest in P7 (Shannon index = 1.73 ± 0.09) and lowest in P4 (Shannon index = 1.33 ± 0.01).

To directly illustrate the effect of soil properties to the iPSB community abundance, the major impact factors (pH and Ca content) was linearly analyzed with *pqq*C gene abundance (Fig. 2c,d). Results showed both factors have strongly impacts on iPSB community abundance (*P* = 0.003 or < 0.001). The correlations between the environmental variables and the iPSB communities are presented in Table 2 and S3. pH and Ca and Mg contents were the most highly correlated with the relative abundance and diversity of iPSB communities and with the abundances of *Arthrobacter*, *Bacillus* and *Burkholderia* (Table 2). They were also significantly correlated with AP rather than total P content. The pH was significantly related (*P* < 0.001 or *P* < 0.002) with the relative abundance of genus *Arthrobacter*, *Bacillus*, *Burkholderia* and *Rhodanobacter* (Fig. S4).

**Table 2:** Spearman’s correlation analysis between soil properties, features of the iPSB community and abundance of some iPSB genera.

	P content	AP content	RA_pqqC_	SDI	Arthrobacter	Bacillus	Burkholderia	Rhodanobacter
pH	0.890	**−0.549** ^*^	**0.665** ^**^	**0.720** ^**^	**0.897** ^**^	**−0.698** ^**^	**−0.991** ^**^	**−0.808** ^**^
C	**0.668** ^**^	−0.032	−0.006	**0.537** ^*^	0.290	−0.150	−0.340	−0.214
N	−0.343	−0.150	−0.448	0.299	−0.079	0.121	0.168	0.372
P	1.000	0.446	0.045	0.226	−0.054	−0.025	−0.066	−0.266
AP	0.446	1.000	**−0.553** ^*^	0.482	**−0.695** ^**^	**0.564** ^*^	**0.533** ^*^	0.092
Ca	−0.021	**−0.636** ^*^	**0.697** ^**^	**0.766** ^**^	**0.987** ^**^	**−0.725** ^**^	**0.877** ^**^	**−0.671** ^**^
Mg	−0.054	**−0.821** ^**^	0.418	**0.718** ^**^	**0.772** ^**^	**−0.704** ^**^	**−0.572** ^*^	−0.248
Fe	0.414	0.157	−0.431	−0.091	**−0.531** ^*^	0.186	0.496	0.488
Al	0.457	0.482	−0.460	−0.414	**−0.722** ^**^	0.407	**0.640** ^*^	0.440

^*^significant at *P* < 0.05; **, significant at *P* < 0.01.

A*P*, available phosphorus; RA_*pqqC*_, relative abundance of *pqqC*; SDI, Shannon diversity index.

**Figure 3:**
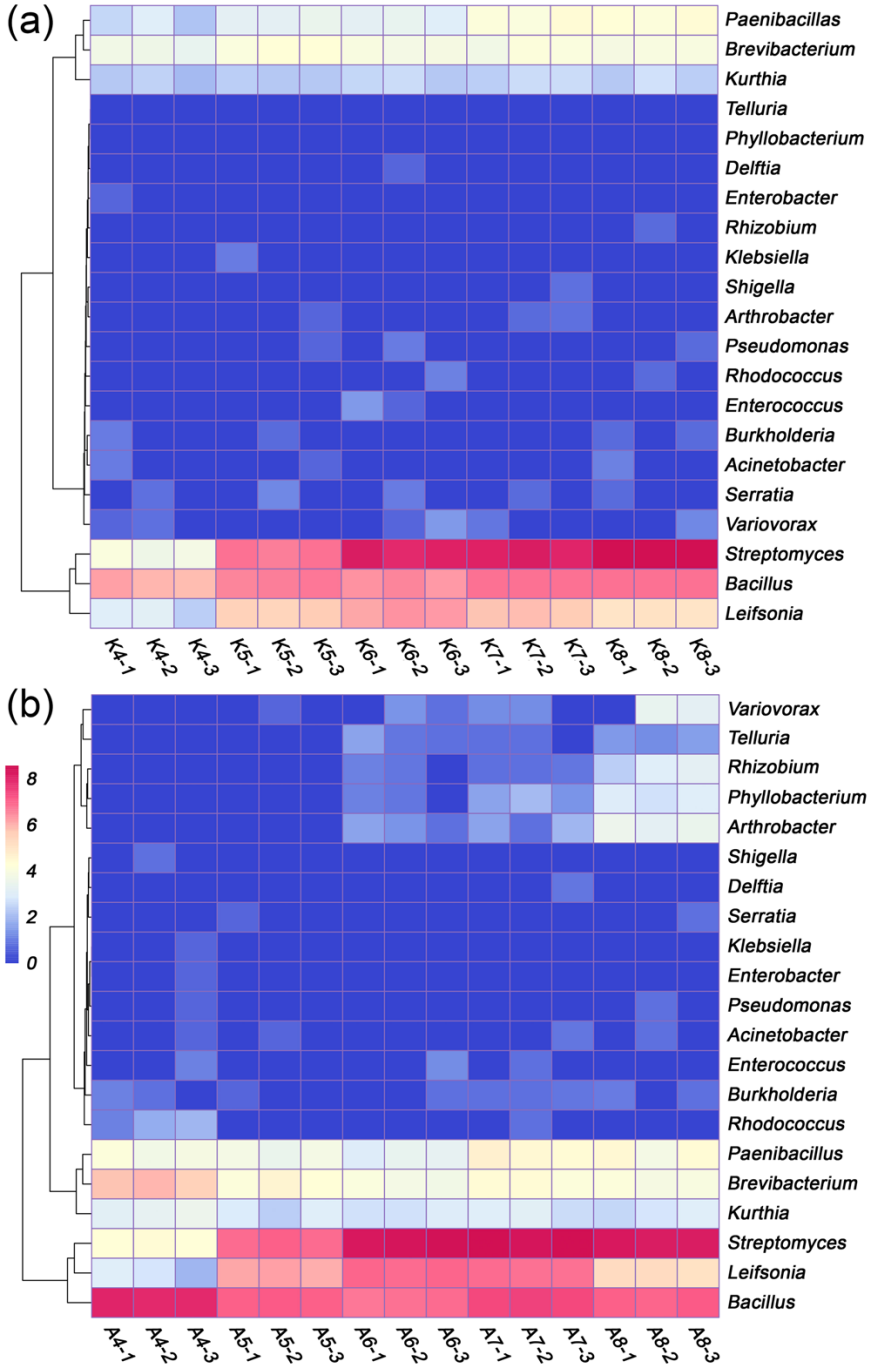
Heatmap analysis of iPSB communities in the pH-adjusted soils based on Bray-Curtis distances. (**a**) Soil P4 with pH adjusted to 4.00 (K4), 5.00 (K5), 6.00 (K6), 7.00 (K7) and 8.00 (K8). (**b**) Soil P8 with pH adjusted to 4.00 (A4), 5.00 (A5), 6.00 (A6), 7.00 (A7) and 8.00 (A8). The plotted values are natural-logarithm transformations of relative iPSB community abundance. The columns are labeled with the soil names and replicate numbers.

The iPSB community structures of the pH-adjusted soils based on database alignment are shown in Fig. 3. *Streptomyces* was the dominant iPSB genus, with an average relative abundance of 87.79 ± 15.56%. *Bacillus* and *Leifsonia* were the next two most abundant genera, with average abundances of 10.62 ± 14.07 and 0.10 ± 0.14%, respectively. The relative abundance of the iPSB communities and the percentage of *Streptomyces* in the alkaline-adjusted soils (K4-K8) increased with the pH (Fig. S5a,c). The highest abundances of the iPSB communities and *Streptomyces* were 16.87 ± 0.34 and 16.81 ± 0.33%, respectively. iPSB abundance (5.01 ± 0.29%) and the percentage of *Streptomyces* (4.90 ± 0.32%) in the acidified soils (A4-A8) were the highest at pH 7 (Fig. S5b,d). The diversity of the iPSB communities increased significantly with pH (*P* < 0.05), except for K5 (Fig. S6a). Diversity did not differ significantly among the iPSB communities in the acidified soils (Fig. S6b).

## Discussions

We assessed the impact of soil pH gradients on population sizes of iPSB communities using qPCR of *pqqC* and on community structure based on database alignment. The *pqq* cluster (including *pqq*FABCDEG genes) was essential for the phosphate solubilizing capacity of many iPSB strains while the mutations of any *pqq* cluster gene may lead to a significant P-mobilizing activity decrease^24^. The using of *pqq*C, the gene expressing for the final catalyst in the production of pyrroloquinoline quinone, has been accepted as the marker gene for tracking microbes able to solubilize inorganic phosphate^23,25^. The application of *pqqC* gene may reflect, in some extent, the size of iPSB community in this study. However, a previous study has indicated that the presence of *pqqC* gene were mainly in Gram-negative bacteria, suggesting that the genus *Bacillus*, including known iPSB species *B. subtilis* and *B. megaterium*, could not be detected by *pqq* gene amplification^37^. This may limit its ecological investigation of iPSB solubilizers. The pyrosequencing of *pqqC* may not reflect the whole structure of iPSB community and the quantification results represented here could not be considered as the whole size of iPSB community. We hence introduced the database alignment method based on 16 S rRNA gene to give more details of the information of iPSB communities.

When soil pH altered, the structure of iPSB community changed accordingly. Some iPSB genera (*Bacillus*, *Arthrobacter* and *Streptomyces*) were always abundant irrespective of soil properties (Fig. 1a and Table S2), in accordance with previous reports that these genera were common in agricultural rhizospheric soil screened on agar plates^38–41^. *Bacillus* was the most abundant genus among the iPSB communities in our study (around 30 ~ 60% abundance), but *Arthrobacter* was reported to dominate iPSB populations in an alkaline soil^25^. The difference may because the *Bacillus* has stronger adaptability under pH changing environments. This could also be found in manually-fixed pH conditions (Fig. 3). The relative abundances of *Arthrobacter* was strongly positively correlated with pH (*P* < 0,0001) while the relation between *Bacillus* and pH was negatively (Fig. S4), which could be explained that they may have different abilities to adapt to changes in environmental pH.

The soil pH gradient is majorly responsible for iPSB community abundance. pH and Ca and Mg contents had the largest effects on iPSB community structure based on the RDA and Monte Carlo permutation verification (Fig. 1b and Table 1). The correlations of these three factors were also highly significant at *P* < 0.01 (data not shown). These results suggest that iPSB strains may have been involved in P desorption from Ca/Mg-P complexes. Both Ca and Mg contents are vitally important for stabilizing soil pH and *vice versa* and have been highly correlated with soil P availability^42–44^. Ca and Mg are also beneficial for bacterial growth and some enzymatic functions such as for zinc-dependent phosphatase^45^. The direct relationship between iPSB populations and Ca or Mg content, however, requires further study. However, the VPA indicated that pH was the primary factor affecting iPSB community structure (Fig. 1c and Table S4). The results differed slightly between the quantification of *pqqC* and the database alignment, but both found that iPSB community abundance increased with pH (Figs 2a and S2), indicating that higher pHs may stimulate the growth of iPSB communities. We tested this suggestion by experimentally increasing the pH with NaOH to determine the effect of alkalization on iPSB communities. The relative abundance and biodiversity of the K4-K8 iPSB communities increased with the pH (Figs S4a and S5a). More interestingly, this phenomenon also occurred in the acidified alkaline soils (A4-A8), where the relative abundance decreased with decreases in pH from 7 to 4 (Fig. S6b). These results suggested that pH was vitally important to the populations of the iPSB communities.

When soil pH increases, the potentials of inorganic P solubilizing capacity of iPSB community would receive enhancement, which could clearly be found in the uptrend of iPSB community abundance along the pH gradients (Fig. 2a). Generally, P is more bioavailable in acidic than alkaline soils, because acidic environments contain more protons for liberating phosphate and slowing the formation of calcic P-bound^13,46^. This latter process is similar to the solubilization of P by iPSB, which solubilize P by secreting organic anions and protons to competitively chelate with metal ions. Acidic soils may thus not need large iPSB communities for releasing P. A large amount of residual P is trapped in alkaline environments primarily by Ca or Mg cations, which may stimulate the growth of iPSB populations for P mobilization, especially under cropping systems^47–49^. The requirement for soil P may therefore account for the increase in iPSB community size along pH gradients.

Predicting phosphate-solubilizing potentials and increasing P use-efficiencies may be based on the correlation between microbiotic structure associated with P and the characteristics of the surrounding environment. Soil pH is an important factor affecting the growth of microorganisms, similar to oxygen pressure and moisture level. Our study demonstrated the dominant role of soil pH on microbial functionality using database alignment and qPCR quantification. The increase in abundance of iPSB communities as a response to soil pH gradients and the optimal diversity at a neutral pH may provide new insights into the modulation of iPSB populations for soil P mobilization.

## Conclusions

Based on database alignment and marker gene quantification methods, our study gave the first report about the iPSB community structure across natural soil pH gradients. The soil pH and Ca content were major factors responsible for structural changes of iPSB communities. Besides, the iPSB community abundance significantly increased with soil pH, which was also verified by manual pH adjustment experiments. The P requirements was considered to be the possible forcing power of iPSB community size.

## Supplementary information

Supporting Information
