# Supplementary material for: Responses to soil pH gradients of inorganic phosphate solubilizing bacteria community
Source: Sci Rep. 2019 Jan 10;9:25. doi: 10.1038/s41598-018-37003-w (PMC6328566; doi:10.1038/s41598-018-37003-w)
Supplement: Supplementary file 1 — Supporting Information [file 41598_2018_37003_MOESM1_ESM.docx]

**Responses to pH gradients of inorganic phosphate solubilizing bacteria community**

Bang-Xiao Zheng^1,2,3,4 #^, Ding-Peng Zhang^5 #^, Yu Wang^6^, Xiu-Li Hao^1,7^, Mohammed A.M. Wadaan^8^, Wael N. Hozzein^8^, Josep Peñuelas^3,4^, Yong-Guan Zhu^1^, Xiao-Ru Yang^1*^

^1^ *Key Laboratory of Urban Environment and Health, Institute of Urban Environment, Chinese Academy of Sciences, Xiamen 361021, PR China*;

^2^ *University of Chinese Academy of Sciences, Beijing 100049, PR China*;

*^3^ Consejo Superior de Investigaciones Científicas (CSIC), Global Ecology Unit, Centre for Ecological Research and Forestry Applications (CREAF)–CSIC–Universitat Autonoma de Barcelona (UAB), Bellaterra, 08193 Barcelona, Catalonia, Spain*;

*^4^ CREAF, Cerdanyola del Vallès, 08193 Barcelona, Catalonia, Spain*;

^5^ *State Key Laboratory of Biocontrol, Key Laboratory of Biodiversity Dynamics and Conservation of Guangdong Higher Education Institutes, College of Ecology and Evolution, Sun Yat-sen University, Guangzhou 510275, PR China*;

^6^ *Key Laboratory of Soil Environment and Pollution Remediation, State Key Laboratory of Soil and Sustainable Agriculture, Institute of Soil Science, Chinese Academy of Sciences, Nanjing 210008, PR China;*

^7^ *Department of Plant and Environmental Sciences, University of Copenhagen, Frederiksberg 1871, Denmark*;

^8^ *Bioproducts Research Chair, Zoology Department, College of Science, King Saud University, Riyadh 11451, Kingdom of Saudi Arabia*.

# Both authors contributed equally.

* Corresponding Author

Key Laboratory of Urban Environment and Health, Institute of Urban Environment, Chinese Academy of Sciences, Xiamen 361021, People’s Republic of China. Phone:(+86) 592 6190560; Fax: (+86) 592 6190977; E-mail: [xryang@iue.ac.cn](mailto:xryang@iue.ac.cn)

Table S1. Geographic information and physical and chemical properties of the soils.

|  | P4 | P5 | P6 | P7 | P8 |
| --- | --- | --- | --- | --- | --- |
| Latitude | 23°16’N  113°35’E | 31°26’N  119°82’E | 23°16’N  113°35’E | 31°64’N  120°74’E | 35°00’N  114°24’E |
| Longitude |  |  |  |  |  |
| Crop cultivation | Maize/Soybean | Wheat/Rice | Maize/Soybean | Wheat/Maize | Wheat/Rice |
| Management | Intercropping | Rotation | Intercropping | Rotation | Rotation |
| Harvest season | June  October | May (Wheat)  October (Rice) | June  October | May (Wheat)  October (Maize) | May (Wheat)  October (Rice) |
| Annual average temperature (°C) | 22.2 | 15.7 | 21.2 | 15.3 | 13.9 |
| Annual precipitation (mm) | 1729.3 | 1109.2 | 1729.3 | 1028.3 | 615.1 |
| pH | 4.60±0.02 e | 5.47±0.15 d | 6.57±0.28 c | 7.55±0.02 b | 8.35±0.01 a |
| Water content (%) | 19.03±1.41b | 32.37±0.12 a | 14.90±0.32 c | 34.30±1.72 a | 16.30±0.56 bc |
| Clay content (%) | 4.04±0.35 a | 1.04±0.09 b | 0.84±0.07 b | 0.80±0.11 b | 1.27±0.04 b |
| Loam content (%) | 44.43±1.81 b | 64.80±3.30 a | 35.81±1.34 c | 44.16±0.88 b | 46.63±1.61 b |
| Sand content (%) | 51.54±2.16 b | 34.17±3.39 c | 63.34±1.36 a | 55.04±0.95 b | 52.10±1.61 b |
| C content (mg g^-1^ DW) | 8.87±0.72 d | 11.61±1.01 bc | 13.45±0.60 b | 26.19±0.24 a | 9.88±0.60 cd |
| N content (mg g^-1^ DW) | 1.20±0.08 d | 1.78±0.05 b | 1.46±0.06 c | 3.00±0.07 a | 0.72±0.03 e |
| NH_4_^+^ content (mg kg^-1^ DW) | 1.20±0.02 a | 0.29±0.07 b | 0.34±0.06 b | 0.32±0.02 b | 0.40±0.17 b |
| NO_x_^-^ content (mg kg^-1^ DW) | 14.93±0.47 a | 21.89±4.99 a | 4.30±1.29 b | 14.97±0.19 a | 2.56±0.08 b |
| P content (mg g^-1^ DW) | 0.56±0.13 b | 0.26±0.15 b | 3.03±0.08 a | 2.22±0.65 a | 0.37±0.04 b |
| AP content (mg kg^-1^ DW) | 19.84±3.15 b | 10.14±1.41 c | 133.98±1.87 a | 8.08±1.30 c | 8.46±1.63 c |
| S content (mg g^-1^ DW) | 3.62±0.04 abc | 3.88±0.44 ab | 2.27±0.16 c | 4.96±0.86 a | 2.79±0.39 bc |
| K content (mg g^-1^ DW) | 12.88±1.49 b | 7.55±0.44 c | 9.52±1.53 c | 18.59±0.11 a | 8.69±0.61 c |
| Ca content (mg g^-1^ DW) | 1.69±0.07 d | 7.23±0.81 c | 5.02±1.31 cd | 16.96±0.75 b | 49.94±2.36 a |
| Na content (mg g^-1^ DW) | 1.77±0.11 bc | 2.03±0.07 b | 1.96±0.18 bc | 2.45±0.08 a | 1.67±0.01 c |
| Mg content (mg g^-1^ DW) | 3.92±0.69 d | 5.48±0.25 c | 3.03±0.35 d | 15.77±0.16 a | 12.93±0.73 b |
| Fe content (mg g^-1^ DW) | 69.20±6.69 a | 39.46±0.58 bc | 46.81±5.62 b | 64.23±1.38 a | 31.10±1.79 c |
| Al content (mg g^-1^ DW) | 169.65±6.93 a | 51.57±0.85 c | 107.32±15.92 b | 99.70±1.06 b | 37.26±2.58 c |

DW, soil dry weight; AP, available phosphorus.

Different letters within a row indicate significant differences at *P* < 0.05.

Table S2. Counts of aligned OTUs (>99% similarities) identified as iPSBs at the genus level for each of the three soil replicates.

| Aligned iPSB genus | P4-1 | P4-2 | P4-3 | P5-1 | P5-2 | P5-3 | P6-1 | P6-2 | P6-3 | P7-1 | P7-2 | P7-3 | P8-1 | P8-2 | P8-3 |
| --- | --- | --- | --- | --- | --- | --- | --- | --- | --- | --- | --- | --- | --- | --- | --- |
| *Acinetobacter* | 0 | 1 | 0 | 0 | 0 | 0 | 6 | 2 | 2 | 0 | 0 | 0 | 0 | 0 | 0 |
| *Arthrobacter* | 54 | 23 | 33 | 316 | 186 | 218 | 38 | 90 | 641 | 170 | 361 | 297 | 319 | 916 | 638 |
| *Bacillus* | 1032 | 516 | 714 | 708 | 811 | 752 | 570 | 1307 | 2144 | 151 | 343 | 316 | 284 | 646 | 325 |
| *Brevibacterium* | 77 | 65 | 47 | 85 | 149 | 162 | 15 | 51 | 235 | 42 | 40 | 67 | 92 | 231 | 122 |
| *Brevundimonas* | 0 | 0 | 0 | 0 | 0 | 0 | 0 | 0 | 0 | 0 | 0 | 0 | 0 | 1 | 0 |
| *Burkholderia* | 119 | 56 | 76 | 39 | 21 | 17 | 16 | 23 | 31 | 5 | 5 | 7 | 0 | 0 | 2 |
| *Chryseobacterium* | 0 | 0 | 0 | 0 | 0 | 0 | 11 | 1 | 27 | 0 | 0 | 0 | 0 | 0 | 0 |
| *Delftia* | 0 | 1 | 1 | 0 | 0 | 1 | 0 | 0 | 0 | 0 | 0 | 0 | 0 | 0 | 0 |
| *Enterobacter* | 2 | 1 | 1 | 0 | 0 | 1 | 131 | 15 | 15 | 6 | 1 | 2 | 1 | 0 | 0 |
| *Enterococcus* | 0 | 0 | 0 | 0 | 0 | 0 | 1 | 0 | 0 | 0 | 0 | 0 | 0 | 0 | 0 |
| *Gordonia* | 0 | 0 | 1 | 0 | 0 | 0 | 0 | 0 | 0 | 0 | 0 | 0 | 0 | 0 | 0 |
| *Klebsiella* | 1 | 0 | 1 | 0 | 2 | 0 | 214 | 5 | 2 | 0 | 0 | 0 | 0 | 0 | 0 |
| *Kurthia* | 5 | 4 | 7 | 6 | 6 | 2 | 0 | 2 | 0 | 1 | 1 | 0 | 1 | 0 | 0 |
| *Leifsonia* | 24 | 2 | 20 | 1 | 0 | 2 | 1 | 13 | 7 | 1 | 1 | 2 | 0 | 1 | 1 |
| *Paenibacillus* | 8 | 7 | 1 | 6 | 4 | 1 | 10 | 16 | 19 | 0 | 2 | 3 | 0 | 6 | 4 |
| *Pantoea* | 1 | 0 | 0 | 2 | 1 | 1 | 1 | 1 | 2 | 0 | 1 | 0 | 2 | 4 | 6 |
| *Phyllobacterium* | 0 | 0 | 0 | 0 | 0 | 0 | 0 | 1 | 1 | 0 | 1 | 0 | 21 | 13 | 21 |
| *Pseudoduganella* | 4 | 1 | 1 | 12 | 12 | 11 | 36 | 47 | 61 | 7 | 7 | 26 | 2 | 2 | 3 |
| *Pseudomonas* | 1 | 5 | 2 | 4 | 22 | 7 | 6 | 4 | 9 | 6 | 89 | 15 | 7 | 13 | 6 |
| *Raoultella* | 0 | 0 | 0 | 0 | 0 | 1 | 0 | 0 | 0 | 0 | 0 | 0 | 0 | 0 | 0 |
| *Rhizobium* | 3 | 6 | 3 | 10 | 9 | 10 | 105 | 61 | 66 | 7 | 9 | 12 | 1 | 4 | 2 |
| *Rhodanobacter* | 37 | 12 | 18 | 18 | 28 | 21 | 6 | 7 | 13 | 3 | 7 | 4 | 0 | 0 | 0 |
| *Rhodococcus* | 4 | 4 | 8 | 4 | 3 | 8 | 3 | 3 | 8 | 4 | 4 | 4 | 1 | 3 | 0 |
| *Serratia* | 2 | 0 | 0 | 0 | 0 | 0 | 6 | 0 | 0 | 0 | 6 | 0 | 0 | 0 | 1 |
| *Shigella* | 0 | 1 | 1 | 0 | 1 | 1 | 0 | 0 | 1 | 0 | 1 | 1 | 0 | 0 | 0 |
| *Sphingomonas* | 0 | 0 | 0 | 0 | 0 | 0 | 1 | 0 | 0 | 1 | 0 | 0 | 0 | 0 | 0 |
| *Staphylococcus* | 0 | 0 | 0 | 0 | 0 | 0 | 0 | 0 | 1 | 0 | 0 | 0 | 0 | 0 | 0 |
| *Streptomyces* | 536 | 130 | 275 | 27 | 50 | 32 | 86 | 359 | 289 | 37 | 44 | 95 | 69 | 164 | 194 |
| *Telluria* | 1 | 0 | 2 | 4 | 9 | 3 | 6 | 8 | 3 | 18 | 6 | 38 | 5 | 51 | 16 |
| *Variovorax* | 1 | 0 | 1 | 11 | 5 | 10 | 0 | 0 | 1 | 24 | 32 | 31 | 3 | 11 | 5 |
| Total | 1912 | 835 | 1213 | 1253 | 1319 | 1261 | 1269 | 2016 | 3578 | 483 | 961 | 920 | 808 | 2066 | 1346 |

Table S3. Spearman’s correlation analysis between some soil properties, features of the iPSB communities and abundances of some iPSB species.

|  | P | AP | RA*_pqqC_* | SDI | *Arthrobacter* | *Bacillus* | *Burkholderia* | *Rhodanobacter* |
| --- | --- | --- | --- | --- | --- | --- | --- | --- |
| WC | -0.118 | **-0.539^*^** | -0.190 | 0.265 | 0.127 | -0.118 | 0.174 | **0.531^*^** |
| Clay | **-0.593^*^** | -0.007 | 0.079 | -0.491 | -0.216 | 0.104 | 0.268 | 0.088 |
| Loam | **-0.750^**^** | **-0.521^*^** | -0.053 | 0.048 | 0.239 | -0.064 | 0.043 | 0.291 |
| Sand | **0.782^**^** | 0.368 | 0.173 | 0.150 | -0.011 | -0.104 | -0.259 | -0.451 |
| N_Amo_ | 0.231 | 0.492 | 0.090 | -0.244 | -0.363 | 0.109 | 0.415 | 0.208 |
| N_Ntr_ | -0.211 | -0.264 | **-0.576^*^** | 0.016 | -0.320 | 0.268 | 0.487 | **0.803^**^** |
| S | -0.046 | -0.396 | -0.107 | 0.265 | -0.023 | -0.104 | 0.274 | **0.522^*^** |
| K | 0.511 | -0.054 | 0.111 | 0.252 | -0.018 | -0.300 | 0.036 | -0.009 |
| Na | 0.256 | -0.261 | -0.420 | 0.200 | -0.033 | -0.007 | 0.003 | 0.261 |

*, significant at *P* < 0.05; **, significant at *P* < 0.01.

AP, available phosphorus; N_Amo_, ammonium N; N_Ntr_, nitrate or nitrite N; RA*_pqqC_*, relative abundance of *pqqC*; SDI, Shannon diversity index; WC, water content.

Table S4. Percentages of variance in iPSB community structure explained by single environmental factors or interactions between two factors.

|  | pH | Ca | Clay | Loam | WC | C | N | N_Amo_ | N_Ntr_ | P | AP | Mg | Fe | Al |
| --- | --- | --- | --- | --- | --- | --- | --- | --- | --- | --- | --- | --- | --- | --- |
| pH | 5.116 | 0.133 | 0.538 | 1.988 | 0.067 | 0.250 | 0.189 | 0.376 | 0.211 | 0.011 | 0.830 | 2.609 | 0.013 | 1.131 |
| Ca |  | 0.955 | 1.543 | 2.028 | 0.924 | 0.202 | 0.039 | 0.586 | 0.017 | 0.414 | 1.600 | 1.260 | 0.131 | 1.454 |
| Clay |  |  | 1.299 | 0.538 | 1.483 | 0.591 | 0.141 | 0.503 | 0.017 | 0.823 | 0.061 | 0.590 | 1.000 | 0.490 |
| Loam |  |  |  | 2.429 | 1.694 | 1.090 | 0.755 | 0.325 | 0.398 | 2.900 | 0.942 | 0.277 | 0.666 | 1.204 |
| WC |  |  |  |  | 0.542 | 0.060 | 0.043 | 0.098 | 0.095 | 0.362 | 2.466 | 0.576 | 0.309 | 0.584 |
| C |  |  |  |  |  | 2.716 | 0.578 | 0.119 | 0.193 | 0.088 | 0.870 | 0.397 | 0.085 | 0.679 |
| N |  |  |  |  |  |  | 1.044 | 0.037 | 0.826 | 0.025 | 0.050 | 1.855 | 0.094 | 0.433 |
| N_Amo_ |  |  |  |  |  |  |  | 0.973 | 0.028 | 0.334 | 0.209 | 0.476 | 0.097 | 0.743 |
| N_Ntr_ |  |  |  |  |  |  |  |  | 0.582 | 0.120 | 0.160 | 0.463 | 0.095 | 0.117 |
| P |  |  |  |  |  |  |  |  |  | 0.627 | 1.264 | 0.394 | 0.255 | 0.597 |
| AP |  |  |  |  |  |  |  |  |  |  | 1.613 | 0.133 | 0.785 | 0.567 |
| Mg |  |  |  |  |  |  |  |  |  |  |  | 3.754 | 1.376 | 0.716 |
| Fe |  |  |  |  |  |  |  |  |  |  |  |  | 1.367 | 3.948 |
| Al |  |  |  |  |  |  |  |  |  |  |  |  |  | 2.069 |

The constraints of sand, S, K and Na contents were automatically aliased by the R program because they were collinear or redundant.

WC, water content; N_Amo_, ammonium N; N_Ntr_, nitrate or nitrite N.


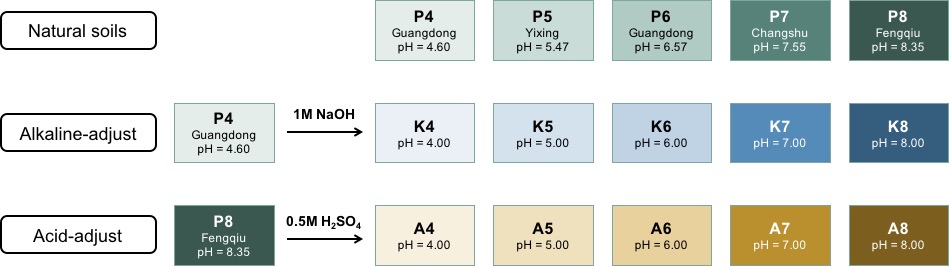


Figure S1. The graphic scheme of manually-pH-adjustment.


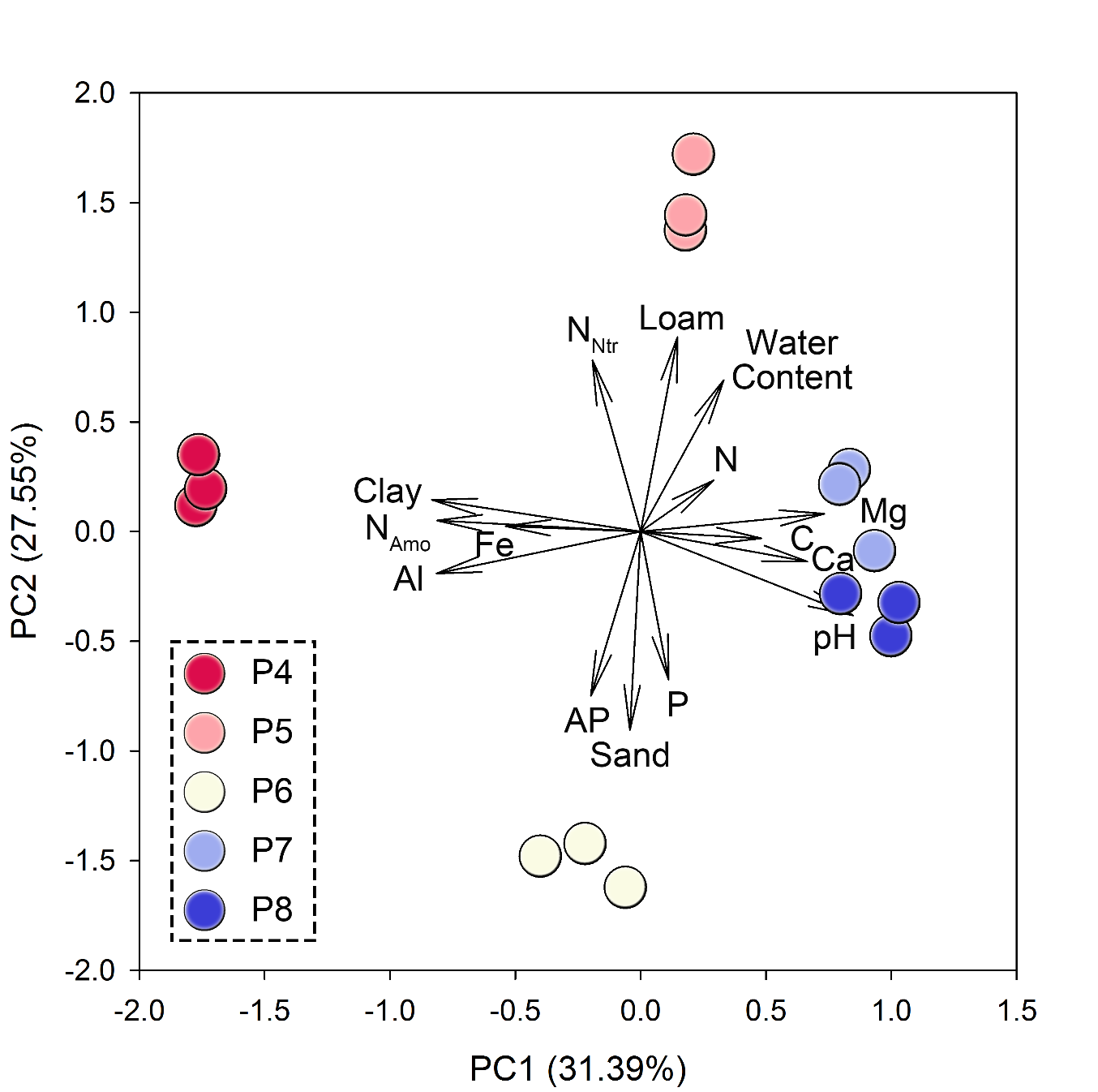


Figure S2. Principle component analysis of the five soil types based on their physical and chemical properties. Results indicated that the physiochemical properties of these five soils were well separated. The two first PCs explained 58.94% of the total variance, with PC1 and PC2 explaining 31.39 and 27.55%, respectively.


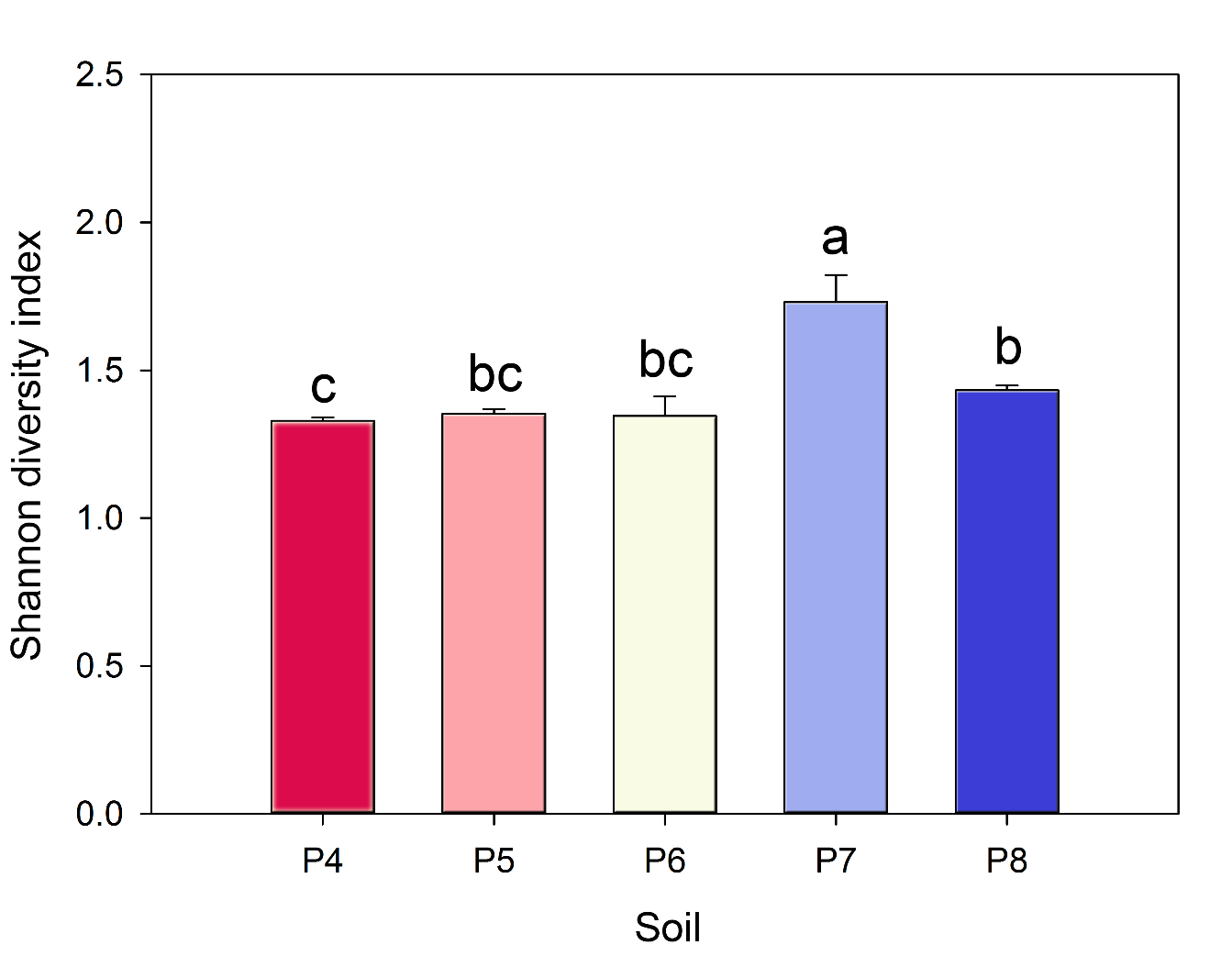


Figure S3. The Shannon diversity index for the soils with different pHs. Different letters above the bars indicate significant differences at *P* < 0.05.


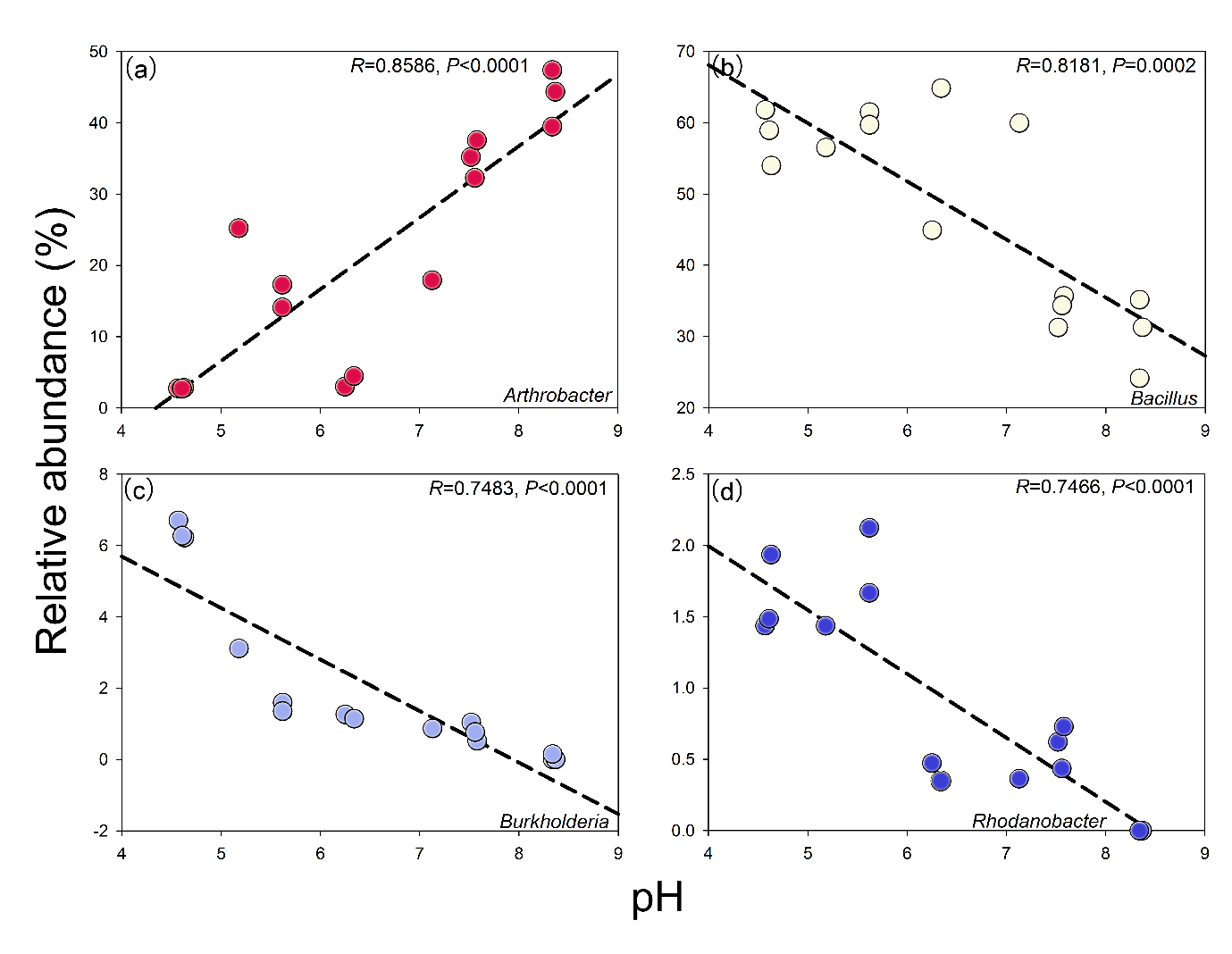


Figure S4. The effect of the pH gradients on the relative abundance of (a) *Arthrobacter*, (b) *Bacillus*, (c) *Burkholderia* and (d) *Rhodanobacter* based on iPSB database alignment.


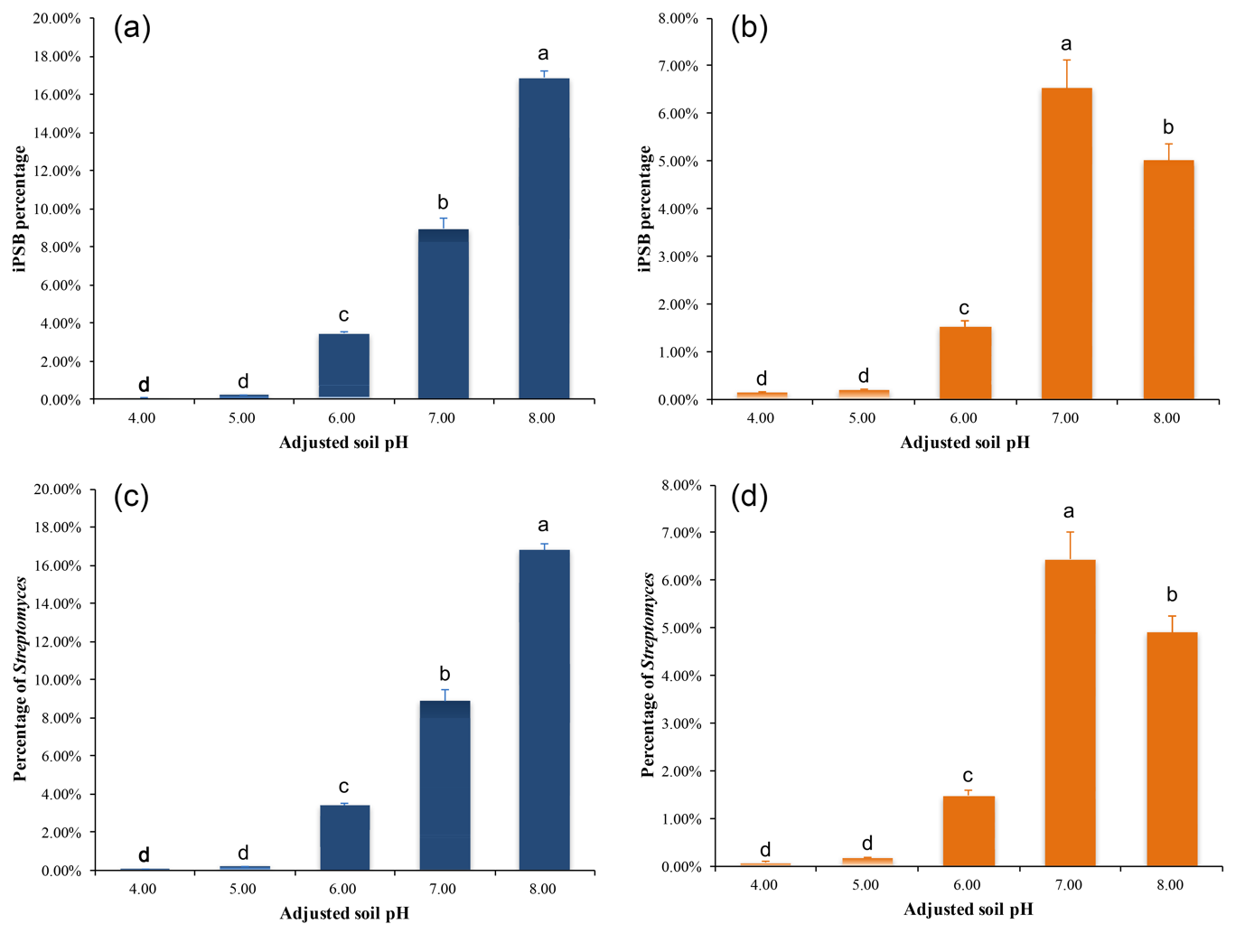


Figure S5. Relative abundance of total iPSB population (a & b) and *Streptomyces* (c & d) in the pH-adjusted soils based on database alignment. The results for the P4 pH-adjusted soils (K4-K8) and the P8 pH-adjusted soils (A4-A8) are shown in a & c and b & d, respectively. Error bars indicate the standard errors of abundance percentage with different letters above showing significant differences at *P* < 0.05.


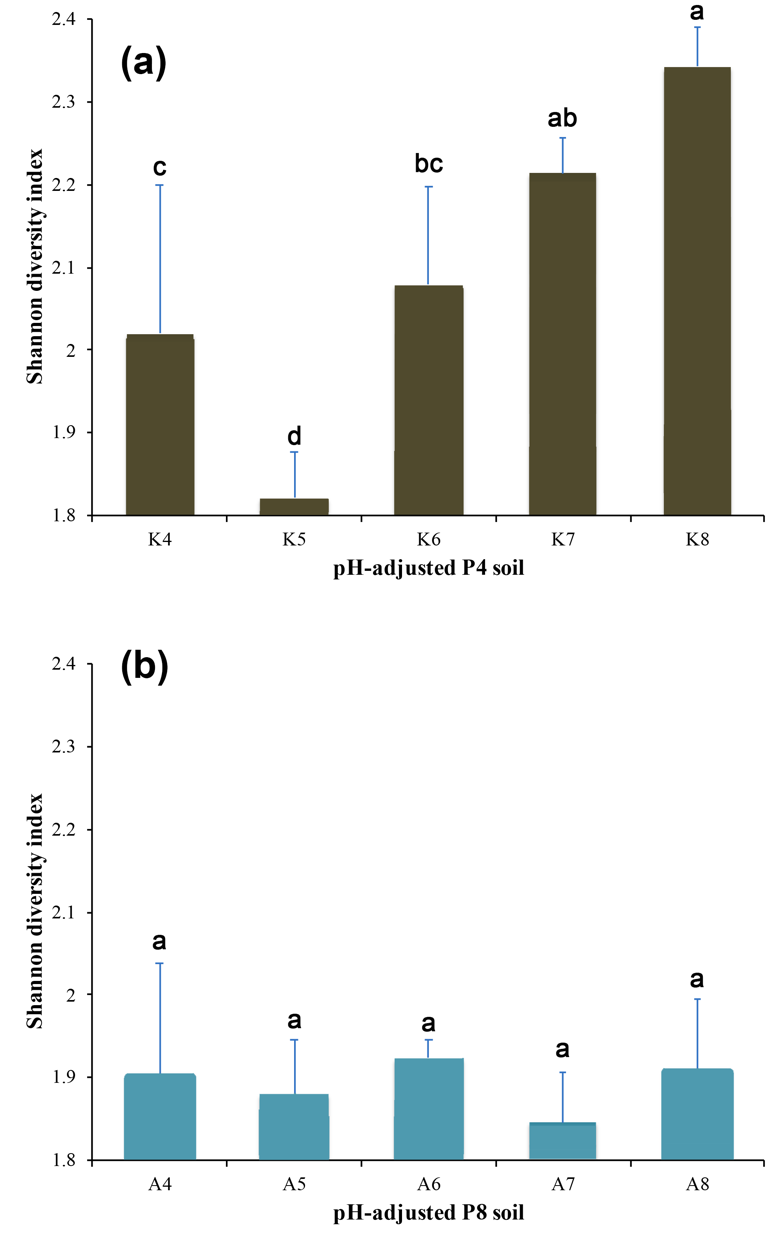


Figure S6. Shannon diversity of the iPSB communities in the pH-adjusted soils. Error bars indicate the standard errors of abundance percentage with different letters above showing significant differences at *P* < 0.05.
